# How to Wedge

**Published:** 1869-10

**Authors:** 


### ARTICLE XIV.

### *How to Wedge.*

The use of the file in the preparation of cavities, is limited to a much smaller number of operations than formerly. Wedging has taken its place to a great extent, and with remarkably good results. The unsightly V shaped spaces between molars and bicuspids are not as common now as formerly. In only rare cases are these openings *now* admissible.

TO GET ROOM BETWEEN THE CENTRAL INCISORS.

If the cavity is cervico-approximal, drive a wedge of orange wood or hickory near the gum. Now drive one, wider than the former, between the crowns. After these have remained a few minutes another may be driven between the crown wedge and the approximal surface of one of the teeth operated upon. The cervical wedge is now loose and a thick and narrow one may be driven in its place to remain during the operation of plugging. If other teeth are to be filled, operations may be commenced upon them, and time allowed for the wedge to swell and the teeth to separate. In this way a great deal of space may often be obtained, even when all the incisors are in contact. If there is already space between the lateral incisors and canines we may wedge between the lateral and central incisors first and then wedge the centrals.

Caution.—There is more *danger* of wedging between the centrals than between any other teeth in the mouth. Especially in *young* subjects is this the case. For it must be remembered that there is a suture between the palatal bones in the median line, which may be forced open, and grave consequences ensue.

Frail teeth must be tenderly treated, and softer wood used than when the teeth are stronger.

Wedging may be applied to the molar teeth by using hickory, and cutting the wedge half off before driving it; for the portion to be driven can be bent at an angle and thus made to enter the place desired, when a straight stick could not be.

Many practice the slow process, using cotton, rubber or wood, removing the wedge every day or two.

My experience is in favor of rapid wedging. By the slow process there is often a great deal of pericementitis and periostitis, which of course must be combatted with appropriate remedies.

By the rapid process there is some immediate pain, but it passes away even before the wedges are removed, and any slight soreness of the teeth afterwards may be removed as in the former mode. Tincture of arnica may be applied to the cervical portions of the teeth after the wedges have been removed, and thus come in direct contact with the periosteum of the alveolar process, and the pericementum.

Two or three small doses of mercury, say a fiftieth of a grain each, will be useful in case there is inflammation of periosteal tissue from either mode of wedging. C.
*Missouri Dental Journal.*

